# Proteomics Investigations of Potential Protein Biomarkers in Sera of Rabbits Infected With *Schistosoma japonicum*

**DOI:** 10.3389/fcimb.2021.784279

**Published:** 2021-12-22

**Authors:** Nian-Nian Bi, Song Zhao, Jian-Feng Zhang, Ying Cheng, Chen-Yang Zuo, Gang-Long Yang, Kun Yang

**Affiliations:** ^1^ School of Public Health, Nanjing Medical University, Nanjing, China; ^2^ National Health Commission (NHC) Key Laboratory of Parasitic Disease Control and Prevention, Jiangsu Provincial Key Laboratory on Parasite and Vector Control Technology, Jiangsu Institute of Parasitic Diseases, Wuxi, China; ^3^ The Key Laboratory of Carbohydrate Chemistry & Biotechnology, Ministry of Education, School of Biotechnology, Jiangnan University, Wuxi, China

**Keywords:** *Schistosoma japonicum*, proteomics, label free, biomarker, bioinformatics

## Abstract

Schistosomiasis is a chronic parasitic disease that continues to be a pressing public health problem in many developing countries. The primary pathological damage from the disease is granuloma and fibrosis caused by egg aggregation, and early treatment can effectively prevent the occurrence of liver fibrosis. Therefore, it is very important to identify biomarkers that can be used for early diagnosis of *Schistosoma japonicum* infection. In this study, a label-free proteomics method was performed to observe the alteration of proteins before infection, 1 and 6 weeks after infection, and 5 and 7 weeks after treatment. A total of 10 proteins derived from *S. japonicum* and 242 host-derived proteins were identified and quantified as significantly changed. Temporal analysis was carried out to further analyze potential biomarkers with coherent changes during infection and treatment. The results revealed biological process changes in serum proteins compared to infection and treatment groups, which implicated receptor-mediated endocytosis, inflammatory response, and acute-phase response such as mannan-binding lectin serine peptidase 1, immunoglobulin, and collagen. These findings offer guidance for the in-depth analysis of potential biomarkers of schistosomiasis, host protein, and early diagnosis of *S. japonicum* and its pathogenesis. Data are available *via* ProteomeXchange with identifier PXD029635.

## Introduction

Schistosomiasis is a chronic zoonotic parasitic disease that affects at least 78 countries and regions, and hundreds of millions of people are at risk of infection (LoVerde, 2019). Thus, it is of great public health significance. *Schistosoma japonicum* is one of the pathogens of human schistosomiasis, which is mainly distributed in Asia, in countries such as China, the Philippines, and Indonesia (Gryseels et al., 2006; McManus et al., 2018). The pathological damage caused by *S. japonicum* is mainly liver and portal vein system damage (Carson et al., 2018; Hu et al., 2020). After mating, paired schistosomes residing in the mesenteric vein could produce a large number of eggs that accumulate in the liver and result in liver granulomas, progressing to liver fibrosis (McManus et al., 2018). Even if the adults in the host are eliminated, the serious pathological damage caused by granulomas is difficult to reverse (Hu et al., 2020), and therefore, identifying and testing potential candidate biomarkers for the treatment of schistosomiasis is of high priority.

At present, the gold standard for the diagnosis of *S. japonicum* continues to be the Kato–Katz method (McManus et al., 2018), in which fecal smears are examined under a microscope for the presence of eggs. Other diagnostic methods, such as serological diagnosis, detect soluble egg antigens (McManus et al., 2018; Hu et al., 2020) by antigen–antibody reaction, and molecular biological methods detect *S. japonicum* nucleic acid fragments (Weerakoon et al., 2017) by polymerase chain reaction (PCR). However, due to the limitations of sensitivity, specificity, cost, and field application, it is difficult for these methods to achieve early diagnosis of *S. japonicum* infection. Additionally, it is difficult to distinguish whether the infection is active or not (Gryseels et al., 2006; Bergquist et al., 2009). With the migration of blood in the host, *S. japonicum* secretes excreta into the blood (Deelder et al., 1980) when feeding on red blood cells. The origin of circulating cathodic antigen and circulating anodic antigen is that it has a positive or negative charge at neutral pH according to its electrophoretic mobility which is called CCA and CAA (Casacuberta et al., 2016). They can be detected by enzyme-linked immunosorbent assay (ELISA) or lateral flow analysis based on monoclonal antibodies (McManus et al., 2018). These two antigens are markers of active infection of *Schistosoma* and can be detected (van Dam et al., 1996) when they are not spawned in the early stage of infection, and therefore, they can be used as potential serum biomarkers after infection.

The development of proteomics technology has enabled researchers to quickly obtain a large amount of human physiological and pathological information (Karczewski and Snyder, 2018) by evaluating biomarkers to distinguish diseases and determine the rules of pathological changes (Bojkova et al., 2020; Puray-Chavez et al., 2021). The proteomics related to *S. japonicum* mainly focus on secretory proteins, parasites, and proteomics at various developmental stages (Cheng et al., 2005; Liu et al., 2009; Hong et al., 2013; Cao et al., 2016a; Cao et al., 2016b; De Marco Verissimo et al., 2019; Li et al., 2020), while there have been relatively few studies on the changes in the host during the disease (Shiff et al., 2006). In addition, the entire proteome of unisexual infected female worms (FSS) and bisexual infected mature female worms (FMS) of *S. japonicum* was studied by iTRAQ and liquid chromatography–tandem mass spectrometry (LC-MS/MS) techniques. The differentially expressed proteins (DEP) were identified and analyzed, and the role of DEPs in sexual maturation and reproductive development of *S. japonicum* (Li et al., 2020) was further analyzed.

Serum, as a repository of secreted proteins from organs of the entire body, reflects the pathological and physiological changes in the body, which may include biomarkers of disease and treatment (Geyer et al., 2017). Because *S. japonicum* excretes secretory proteins (Deelder et al., 1980) in the host, there may be parasitic or host-related biomarkers in serum proteins after infection. Kardoush et al. (2016) identified a large number of candidate hosts and parasite biomarkers of acute and chronic schistosomiasis in mice by combining surface-enhanced laser desorption ionization–time of flight mass spectrometry (SELDI-TOF MS), matrix-assisted, laser desorption and ionization (MALDI-TOF MS), and Orbitrap techniques in the serum proteome of *Schistosoma mansoni*, and they verified the protein glutathione S-transferase (GST:25KDa), which can be used as a potential biomarker for early diagnosis. Onile et al. (Onile et al., 2017) selected proteomic methods to find several parasite- and host-specific protein biomarkers in a clinical urine sample cohort, which may have diagnostic potential in urine detection of schistosomiasis and schistosomiasis-related bladder cancer in Egypt. Proteomics has been successfully used in the testing and identification of biomarkers for a variety of diseases. It is an effective means to explore and verify the differences in the occurrence and development of diseases by combining mass spectrometry and bioinformatics.

In this study, a label-free proteome shotgun method was used to screen differential proteins in the serum after infection with *S. japonicum* and treatment. Then, we explored potential biomarkers by dynamic protein expression changes occurring in the host proteome in response to *Schistosoma* infection and treatment.

## Material and Methods

### Ethics Statement

All animal experiments were conducted in strict accordance with the guidelines of the International Association for Assessment and Accreditation of Laboratory Animal Care (IAAALAC). The protocol was approved by the Ethical Review Committee of Jiangsu Institute of Parasitic Diseases (JIPD) [permit ID JIPD-2020-013].

### Pretreatment of Serum Protein Samples

In this experiment, four New Zealand rabbits (n = 4), weighing 2.0–2.5 kg, were used as experimental subjects to observe the changes in proteins before infection, after infection, and after treatment. First, New Zealand rabbits were infected by abdominal patch with 1,000 cercariae of *S. japonicum*. The cercariae were provided by the Jiangsu Institute of Parasitic Diseases. All rabbit stool samples were examined by the miracidium hatching test (Yu et al., 2007; He et al., 2018), and the presence of miracidia confirmed the success of infection. The serum was collected before infection (Ctrl group) as a negative control (Guo et al., 2012) as well as 1 week (E1w) and 6 weeks (E6w) postinfection. After 7 weeks of infection, praziquantel (PZQ) (150 mg/kg) was orally administered for 2 days, and the serum was collected at 5 weeks (T5w) and 7 weeks (T7w) after treatment. No adults were found in the portal system 16 weeks posttreatment after sacrificing the rabbits by humane methods. The peripheral blood was collected through the ear vein.

After the samples were collected, the serum was separated in a thermostat at 37°C for 4 h. The serum was centrifuged for 5 min at 200×g and then stored at -80°C. For the serum sample preparation of each group, equal volumes of four biological repetitive serum samples were pooled into one group (Schwamborn et al., 2009), from which three technical repeats were produced. A volume of 60 μl of pooled serum sample from each group was processed using the ProteoExtract Albumin/IgG Removal Kit (Merck Millipore, Billerica, MA, USA). According to the commercial instructions for the kit, albumin and IgG in high abundance were removed to reduce the complexity of the serum (Xing et al., 2019). After removal of high-abundance proteins, the serum protein was concentrated in a 10-kDa centrifugation ultrafiltration tube (Amicon Ultra-0.5, Millipore, MA, USA), and the concentrated protein was obtained by 13,000 ×g centrifugation for 5 min. Using a BCA kit (Beyotime, Shanghai, China) and bovine serum albumin (BSA) as the standard, a standard curve was determined by SpectraMax i3 (Molecular Devices, USA) at 562 nm, and the concentration of the sample was calculated.

### Digestion of Serum Proteins

Serum proteins were denatured in 8 mM urea/40 mM NH_4_HCO_3_ buffer with protease inhibitors (100×; Beyotime, Shanghai, China). Proteins were added with 10 mM dithiothreitol (DTT) for 60 min at room temperature (RT) and then alkylated by 20 mM iodoacetamide (IAM) for 45 min in the dark at RT. The solution was buffer-exchanged with 40 mM NH_4_HCO_3_ to dilute 8-fold with a 10-kDa centrifugation ultrafiltration tube.

According to the manufacturer’s instructions, proteolytic digestion was performed by the addition of 30 μl Sequencing Grade Modified Trypsin (Promega, Madison, WI) (enzyme:protein ratio of 1:50 w/w) and incubation while shaking at 500 rpm and 37°C for 12 h. Then, 7.5 μl trypsin was added so that the final ratio was 1:40 trypsin, with incubation at 37°C for 4 h. After restriction endonuclease digestion, 50% formic acid (FA) (v/v) was used for acidification, and dilution with 0.1% FA (v/v) was performed to terminate the trypsin reaction, with subsequent centrifugation at 13,000 ×g for 15 min. The supernatant was collected, and the peptide concentration was measured using the NanoDrop 2000c spectrophotometer (Thermo Scientific, USA).

The polypeptide (Yang et al., 2018) was purified by C18 solid-phase extraction. First, 50 μg peptide solution was transferred to the internally prepared tip column. The tip column was cleaned with acetonitrile (ACN), 0.1% FA/50% ACN (v/v), and 0.1% trifluoroacetic acid (TFA) (v/v). After injection, the sample was washed with 1% FA (v/v). Finally, the sample was collected with 0.1% FA/50% ACN (v/v). Appropriate volumes of the peptides (three independent replicates from five groups) were evaporated to dryness using a freeze-dryer (Labconco, USA).

### LC-MS/MS Analysis

Freeze-dried peptides were suspended in 0.1% FA/2% ACN (v/v) before MS analysis. The peptide was diluted to 0.5 μg/μl, and the suspension was vortexed for 1 min and centrifuged for 10 min at 13,000 × *g*. The Easy-nLC 1200 system (Thermo Science, San Jose, CA, USA) and high-resolution Orbitrap Fusion Lumos spectrometer (Thermo Science, San Jose, CA) were used for analysis. Each injection volume was 3 μl. The samples were first separated on an Easy-nLC 1200 system with an RSLC C18 column (1.9 μm × 100 μm × 20 cm). The mobile phase buffer A is water, and the buffer B is 90% ACN. The flow rate is set to 550 nl/min. The data-dependent high-energy collision dissociation (HCD) fragmented the 20 most abundant ions. The spray voltage was in static mode. The spectrum (the AGC target was 4.0e5, and the maximum injection time was 50 ms) was collected in the range of 350–2,000 nm pulse z with a resolution of 60,000, followed by data-related higher-energy collisional dissociation (HCD)-MS/MS (resolution of 30,000, collision energy of 34%, step collision energy of 5%, target of 5.0e4, maximum injection time of 35 ms, microscan 1). Charge state screening can reject single charge ions and ions with more than eight charges. A dynamic exclusion time of 45 s was used to distinguish newly selected ions from previously selected ions.

### Statistical Analysis of Mass Spectrometry

All the original MS data were processed using MaxQuant software (version 1.6.14.0) and its built-in search engine Andromeda, and the databases for *S. japonicum* and New Zealand rabbits from UniProt (downloaded on 09/23/2020) were used for peptide recognition and protein inference. The false discovery rate (FDR) at both the peptide and protein levels was set at 1%. The key settings were digestion enzyme: trypsin/p, and tolerated missed cleavage: 2. Carbamidomethyl (C, +57 Da) was set as fix modification, and Oxidation (M, +16 Da) modification was included as a variable modification. In other LC-MS operations, peptide identification was converted to unidentified characteristics based on the quality of the match and the retention time of the run-to-run recalibration (the “match between run” option in MaxQuant). Label-free quantification (LFQ) using MaxQuant requires at least one peptide ratio (Cox et al., 2014) for each identified protein, based on the determination of the median paired common peptide ratio between samples. The LFQ value for MaxQuant was imported into Perseus software (version 1.6.14.0). The proteins were initially filtered with “potential contaminant” and “reverse.” Proteins identified with at least one unique peptide were considered for further data analysis. Samples were then grouped into experimental and Ctrl groups, and only proteins with two valid values in at least one group were retained.

Based on the assumption that the strength of most peptides was constant between samples, the peptide strength was normalized to minimize the overall proteome difference. The logarithmic conversion of proteome LFQ intensity was performed to reduce the effect of outliers. The missing value was replaced by a random value generated by the Gaussian distribution to simulate the low-abundance LFQ value, and each sample in the distribution with a width of 0.3 and a downward shift of 1.8 was calculated. The logarithmic ratio (fold change) was calculated according to the difference in the average log_2_ LFQ intensity between the experimental groups and the Ctrl group. The proteins that were significantly up- or downregulated (two-sided, unpaired Student’s *t*-test with equal variance assumed, p-adjust< 0.05, n = 3, | log_2_FC | > | log_2_1.5 |) in at least one infected sample compared with the corresponding Ctrl are shown. The data were standardized using Z-scoring before row-wise clustering and plotting. In the statistical test, double-tailed, unpaired, and equal-variance Student’s *t*-test calculations using Benjamin & Hochberg correction were carried out using LFQ data. Statistical analysis and visualization of differential expression were performed by hierarchical clustering and principal component analysis (PCA). Heat maps and PCA maps were generated by Pheatmap (R packet), status (R packet), and ggplot2. The host differential proteins were enriched by GO function using the cluster profiler (R package). The proteins identified as those of *S. japonicum* provide BLAST results of protein sequences between *S. japonicum* and rabbit to ensure the results and underwent GO function annotation (Conesa et al., 2005; Sotillo et al., 2015) by Blast2GO. The mass spectrometry proteomics data have been deposited to the ProteomeXchange Consortium *via* the PRIDE (Perez-Riverol et al., 2019) partner repository with the dataset identifier PXD029635. 

## Results

### The Host Proteome Changed After Infection and Treatment

**Figure 1:**
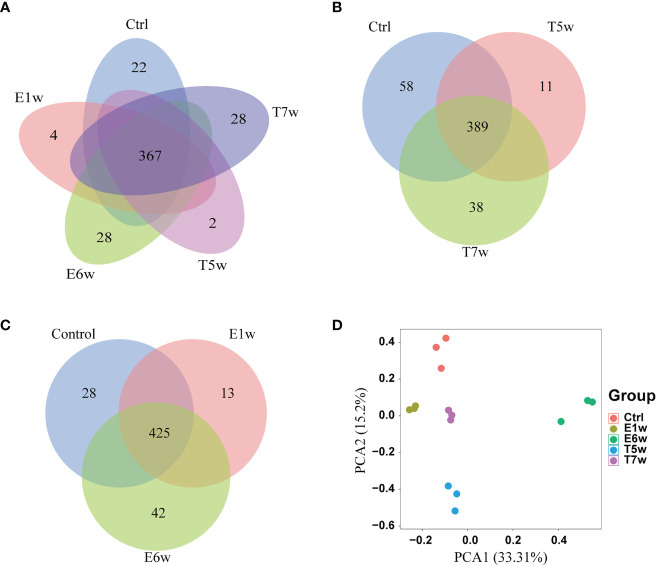
Host proteins changed after *Schistosoma japonicum* infection and treatment. **(A)** Venn diagram of the identified host proteins in the five groups, Ctrl, E1w, E6w, T5w, and T7w. **(B)** Overlap proteins after treatment. **(C)** Overlap proteins after infection. **(D)** PCA analysis of host proteins.

After infection and treatment, five groups of samples (Ctrl, E1w, E6w, T5w, and T7w) were analyzed to identify candidate biomarkers for infection and treatment of schistosomiasis. In this study, a total of 682 proteins were identified using a label-free shotgun proteomics approach. Nearly all the identified proteins (98.53%) were from host samples, and only 11 proteins were uniquely derived from *S. japonicum*. For the host, a total of 671 proteins were identified from the Ctrl, E1w, E6w, T5w, and T7w samples (**Supplementary Table 1**). The Venn diagram in **Figure 1A** shows that 367 proteins were commonly identified in the five groups, in which the number of proteins was 546, 491, 531, 432, and 532, respectively. Regarding proteins identified in only one group, 22 proteins were exclusively identified in the Ctrl group, 4 in E1w, 28 in E6w, 2 in T5w, and 28 in the T7w group. Venn diagrams (**Figures 1B, C**) show the overlaps between the Ctrl, the groups after infection, and the groups after treatment.

To quantitatively analyze the proteins from the five groups and discover potential biomarkers during *Schistosoma* infection and treatment, the intensity of proteins was applied and filtered with the following parameters based on label-free quantitative proteomics: samples were grouped into Ctrl and four experimental groups, and only proteins with two valid values in at least one group were retained. Finally, 396 high-quality proteins were selected for the next steps. The principal component analysis (PCA) highlighted the high degree of reproducibility among three replicates and clearly classified the five groups (**Figure 1D**). PCA1 and PCA2 accounted for 33.31% and 15.2% of the principal components, respectively. It was suggested that the infection of *S. japonicum* and praziquantel treatment obviously affected the protein expression and secretion of the host rabbit. There were differences between the proteomes of the five studied groups.

**Figure 2:**
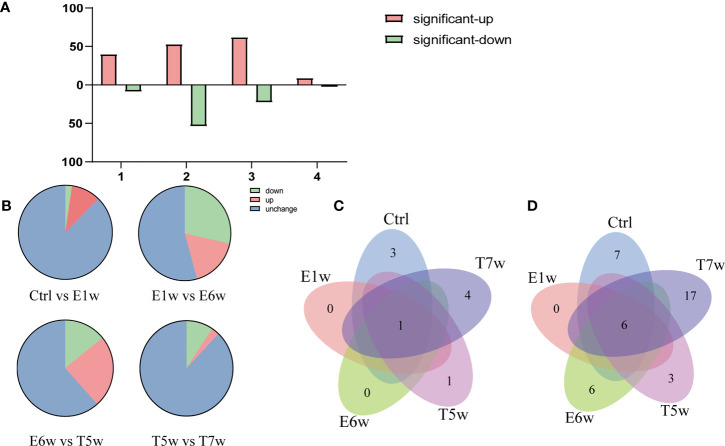
Numbers of upregulated or downregulated DEPs changed when comparing four experimental groups **(A)** with the Ctrl group and **(B)** between two consecutive groups. **(C)** Venn diagram of the identified *Schistosoma*-derived proteins searched against a combination database of rabbit and *S. japonicum* in the five groups, Ctrl, E1w, E6w, T5w, and T7w. **(D)** Venn diagram of the identified *Schistosoma*-derived proteins searched *S. japonicum* database only in the five groups, Ctrl, E1w, E6w, T5w, and T7w.

Of note, methods revealed that the E6w group differed the most from the other groups, indicating that the protein expression profile encountered a major shift in the E6w group. Furthermore, we compared the protein expression between the four experimental groups and the Ctrl group (**Figure 2A**), as well as two consecutive groups (**Figure 2B**). It was found that a total of 242 proteins significantly changed after infection or treatment. Compared with the Ctrl, the differential protein increased over time, reached a peak at 6 weeks after infection, and then began to decrease after treatment.

### Identification of *S. japonicum*-Derived Protein in the Host Serum

A total of 11 *Schistosoma* unique proteins in host serum were identified when the raw data were searched against a combination database of rabbit (taxid:9986) and *S. japonicum* (taxid:6182). Considering that there was suppression by highly abundant proteins in the MS identification, the data were also searched against the *S. japonicum* (taxid:6182) database only, as described in a previous study (Onile et al., 2017), and 45 *Schistosoma* proteins were identified (**Supplementary Table 2**). Therefore, the search results for the two methods were combined for further analysis of proteins derived from *S. japonicum*.

Venn diagrams were generated to identify proteins unique to each group and the overlaps of identified *S. japonicum* proteins. Out of 11 Schistosoma proteins searched against a combination database of rabbit and *S. japonicum*, 3, 0, 0, 1, and 4 proteins were unique to Ctrl, E1w, E6w, T5w, and T7w, respectively, and 1 protein was found common to all studied groups (**Figure 2C**). When searching the database of *S. japonicum* only, 7, 0, 6, 3, and 17 proteins were unique to Ctrl, E1w, E6w, T5w, and T7w, respectively, and 6 proteins were found common to all studied groups (**Figure 2D**). Due to many conservative proteins between *S. japonicum* and rabbit, the proteins identified from *S. japonicum* in the Ctrl group were removed. The “unique peptide” of *S. japonicum* identified by blastp through NCBI using default settings (Park et al., 2009), and the “organism” contained rabbit (taxid:9986) and *S. japonicum* (taxid:6182). According to the maximum of “MaxScore,” the “scientific name” of proteins distinguished the proteins of *S. japonicum* or rabbit. Ten proteins derived from *S. japonicum* include tubulin, calmodulin, EF-hand domain-containing protein, and SJCHGC06596 protein, etc. It has been predicted that they have molecular functions such as calcium ion binding, GTP binding, and DNA binding. The GO terms of the identified *Schistosoma* proteins are summarized in **Table 1**.

**Table 1:** BLAST results between *S. japonicum* and rabbit and Gene ontology analysis of *Schistosoma*-derived proteins.

Sequence	UniProt ID	Description	Max score	E value	Sequence coverage [%]	GO terms
IALEQAR	A0A4Z2CWQ3	Translin-associated factor X-interacting protein isoform 2	24.8	0.45	0.9	F:calcium ion binding
DGVPDIVILVDSGNTQQLQVILR	A0A4Z2CXV5	T-cell immunomodulatory protein	75.3	1.00E-17	3.5	P:integrin-mediated signaling pathway; C:integral component of membrane
DVNAAIATIK	A0A4Z2D895	Tubulin alpha-3 chain	33.3	8.00E-04	32	F:GTPase activity; C:cytoplasm; P:cytoskeleton organization;
EDILYSHLK	A0A4Z2DER5	Phosphatidylinositol 4-phosphate 3-kinase C2 domain-containing subunit alpha	32.9	0.001	0.4	F:kinase activity; P:phosphorylation;
SLQNANVIQTLR	A0A4Z2DIC3	Phosphatidylinositol-binding clathrin assembly protein	40.5	3.00E-06	0.7	F:1-phosphatidylinositol binding; C:cilium; P:clathrin coat assembly
ADQLTEEQIAEFKEAFSLFDKDGDGTITTK	A0A4Z2DTY8	Calmodulin	97.3	8.00E-25	20.8	F:calcium ion binding; C:cytoplasm; P:protein phosphorylation
VTYDGILGNSALEMTK	Q5DCJ5	SJCHGC06596 protein	53.7	2.00E-10	4.5	F:nucleotide binding; C:ribosome;
EAFSLIDQNR	Q5DFN5	EF-hand domain-containing protein	35.4	1.00E-04	9.8	F:calcium ion binding
AVEIKELEGLPGDVLR	A0A4Z2DRZ9	DNA-directed RNA polymerase	52.4	4.00E-10	1.5	F:DNA binding; C:nucleus; P:transcription, DNA-templated;
LSDDEPLLEK	C1LFK2	Peptidase M	34.6	3.00E-04	6.1	C:chromatin; F:double-stranded DNA binding; P:response to UV

B, biological process; M, molecular function; C, cell component.

“Query Cover” and “Per. Ident” are both 100%. Max score: highest alignment score (bit-score) between the query sequence and the database sequence segment; Query Cover: percentage of the query length that is included in the aligned segments. This coverage is calculated over all segments.

### Host Protein Expression Landscape Temporal Analysis

To obtain a temporal change in the host proteins during five points after infection and treatment, we analyzed system-wide differences in protein levels over time. Comparison of host proteins allowed us to dissect the contribution of the *Schistosoma* infection and treatment to changes in the host proteomics. We found that 242 host proteins were differentially expressed in the samples of five groups after correlating their expression with infection and treatment.

**Figure 3:**
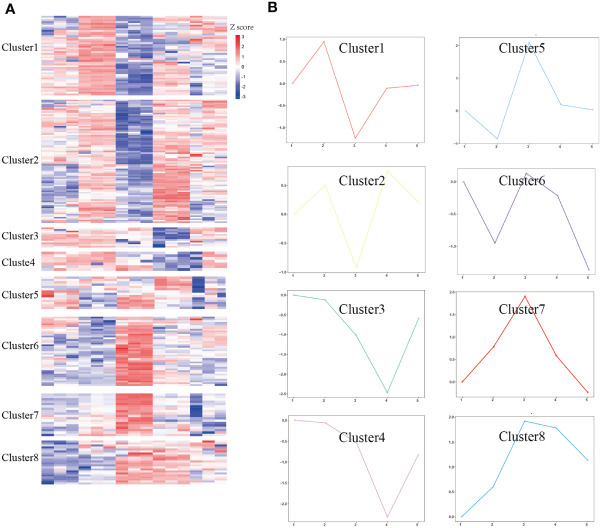
Temporal analysis of DEPs. **(A)** DEPs were clustered by Z-score. **(B)** The log_2_FC of proteins in eight clusters was quantified. The solid colored line represents the average value of the ratio between the experimental group and the control group.

To address our hypothesis that during infection and treatment of *S. japonicum*, some meaningful proteins underwent temporal change, we used HCA analysis to identify protein groups or clusters that demonstrated a similar temporal pattern (**Figure 3A**). Unsupervised clustering defined eight unique clusters that characterized the temporal regulation of the proteome. The k-means method is useful to cluster proteins into a specified number of classes based on the similarity of temporal profiles. We applied the k-means algorithm and manual selection to group all DEPs into eight distinct clusters for identification of potential proteins dysregulated during *Schistosoma* infection (**Figure 3B**). Clusters 3 and 4 represent proteins that are downregulated, and cluster 8 represents proteins that are upregulated, whereas other clusters represent proteins displaying a bimodal expression pattern, such as a decrease in E6w (clusters 1 and 2) or a gradual increase in E6w (clusters 7 and 8). Among eight clusters, two clusters were upregulated at 1 week after infection, and cluster 7 returned to normal levels after treatment. Clusters 5 and 6 were downregulated at 1 week after infection, which increased at 6 weeks after infection, and decreased with the extension of treatment time. Overall, most of the differentially expressed proteins increased following *Schistosoma* infection, with proteins in cluster 2 displaying the greatest fold changes.

### GO Analysis of Different Protein Profiles

**Figure 4:**
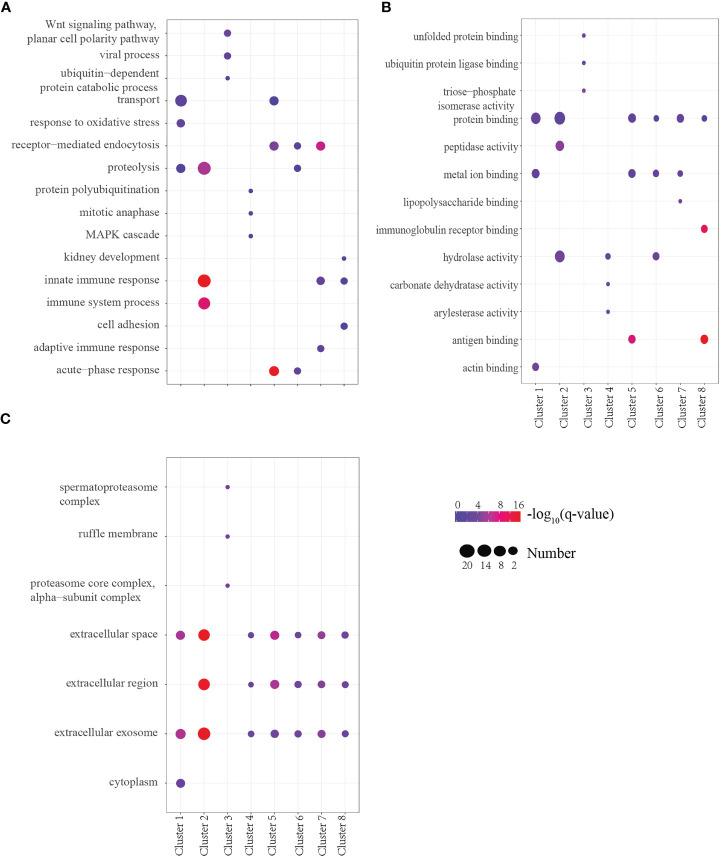
**(A–C)** GO analysis of the top three terms between Biological Process **(A)**, Molecular Function **(B)**, and Cell Component **(C)** in eight clusters.

To determine the biological significance of these DEPs, GO enrichment analysis was performed for every cluster. To obtain a global overview of the types of DEPs, they were also classified into three clusters by gene ontology (GO): cellular component, molecular function, biological process, and protein class. We found that the proteins present in each profile enriched distinct biological processes and molecular function (**Supplementary Table 1**). One of the most rapid changes in early infection was cluster 7, which consisted of 18 proteins that exhibited striking upregulation at E1w postinfection and then tended to downregulate with levels close to Ctrl values by treatment. For the majority of these proteins, the trend was high expression by the E6w group. They are involved in the early stages of immune responses to *Schistosoma* infection. GO analysis indicated that some of these proteins represent innate immune response, receptor-mediated endocytosis, and transport (**Figure 4**).

**Figure 5:**
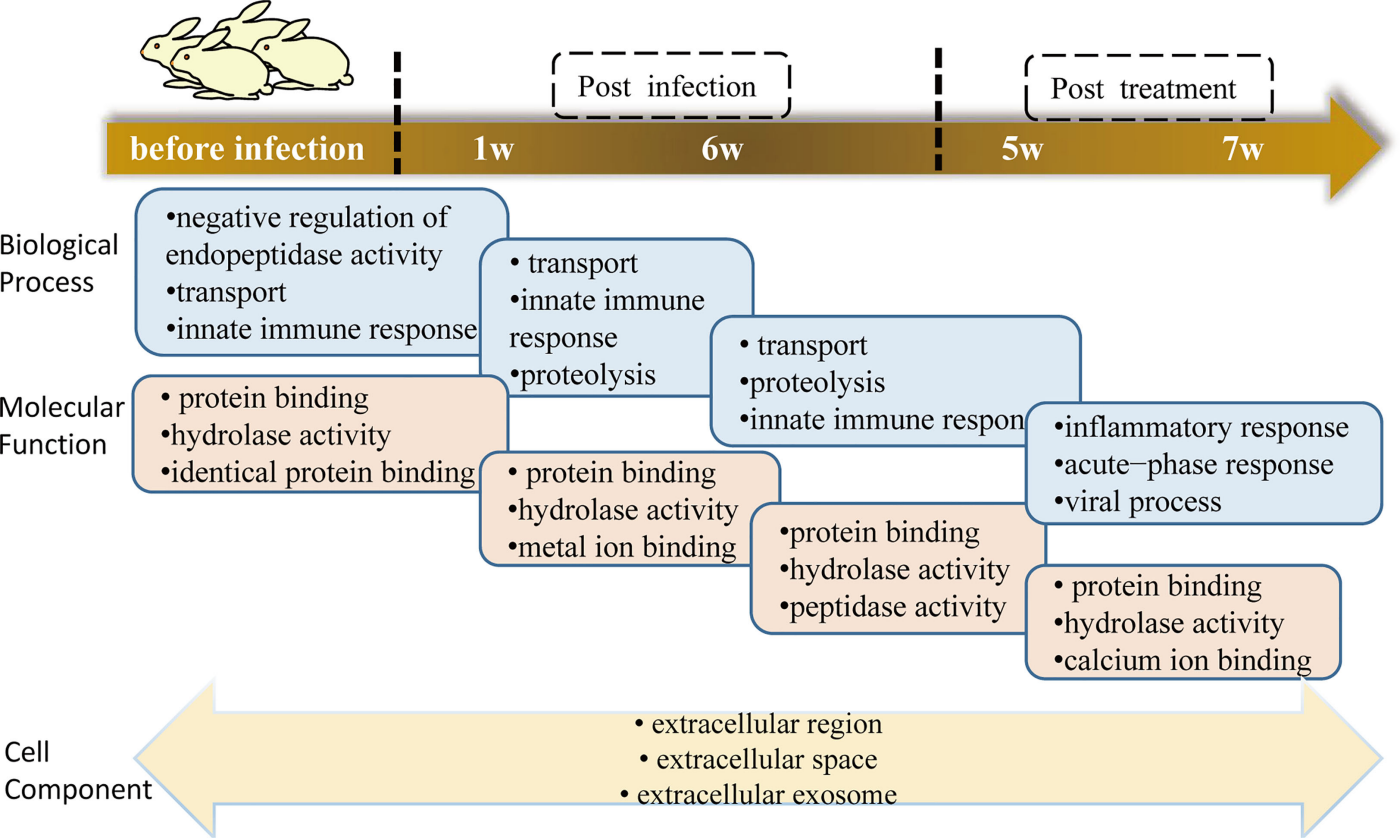
GO terms of DEPs between two consecutive groups.

Proteins annotated to receptor-mediated endocytosis included hemopexin, galectin-3-binding protein, apolipoprotein B, and monocyte differentiation antigen CD14. Proteins annotated to innate immune response included monocyte differentiation antigen CD14, Ig mu chain C region-secreted form, and fibrinogen alpha-chain. Nearly half of these proteins are involved in hydrolase activity, metal ion binding, and protein binding. Among the eight clusters, there were few changes in cellular components, which were concentrated in extracellular exosome, extracellular space, and extracellular region. Only cluster 3 proteins participated in the cytoplasm, extracellular exosome, and nucleus. We enriched the DEPs between each of two consecutive groups into GO terms and found that molecular function differentially changed, but there was no significant difference between cell component and biological process (**Figure 5**). During the first week of infection, there was a negative regulation of endopeptidase activity. With the progress of infection and treatment, the immune system gradually changed from an innate immune response to an inflammatory response and acute-phase response. The cell components of most proteins were enriched in the extracellular region, extracellular space, and extracellular exosome, and most of the molecular functions were protein binding and hydrolase activity.

## Discussion

In this study, the protein expression profile of serum during *S. japonicum* infection and treatment in the animal model was analyzed by label-free quantitative proteomics, and 396 host proteins were further filtered by MaxQuant search. Through these approaches, useful for investigating large numbers of proteins over several time points during infection and treatment, we identified proteins that are likely involved in the infection process in *Schistosoma*. Identifying these proteins in *Schistosoma* samples can provide potential biomarkers for detection and diagnosis. One benefit of our results is that *Schistosoma* infection can be used to discover proteins important in host defense, as parasite modulation of protein expression generally reflects a biological imperative.

*S. japonicum* migrates to various organs through blood and secretes a large amount of protein during the process. CCA and CAA of *S. mansoni* are also excreta such as that produced by *S. japonicum* migration in blood vessels and have been used diagnostically (Deelder et al., 1980; Casacuberta et al., 2016), and therefore, searching for potential biomarkers in serum is a method with diagnostic potential. Due to the low content of these circulating antigens in the host, most of them are mixtures, so the key proteins have not been further studied. Excretory and secretory proteins (ESP) from *Schistosoma* are also a kind of secreted protein with the migration of blood in the host; the supernatant was obtained from different development stages cultured (Mathieson and Wilson, 2010; Cao et al., 2016b; De Marco Verissimo et al., 2019). Through quantitative analysis of the excretory/secretory proteome of the adult (Liu et al., 2009; Hong et al., 2013), it is found that the main components of fatty acid-binding proteins and heat shock proteins HSP70, HSP90, and HSP97 constitute the largest protein family in the ES proteome, which means that these proteins play a central role in immune regulation in the host relationship. ESPs also included actins, tubulins, and 14-3-3. In the present study, 10 proteins derived confidently from *S. japonicum*, including tubulin, calmodulin, EF-hand domain-containing protein, and SJCHGC06596 protein. Proteins with high homology with rabbit such as actin 5c, Histone H4, and HSP 70 were also detected and are highly abundant. HSP70 participates in egg skeleton formation (Dewalick et al., 2011), while HSP70, 90, and actin still exist in isolated exosomes (Zhu et al., 2016). A protein chip method was used to predict the interaction of the three heat shock proteins SjHSP40, SjHSP70, and SjHSP90 with SjSTIP1 through bioinformatics, together with SjPPase as a drug or vaccine target, which has potential diagnostic or therapeutic value (Chen et al., 2014). Histone H4 and H2 were also identified in the serum after infection. Histone and histone-modified proteins have great influence on H4 and H2 in parasites with complex life cycles and multiple developmental stages (Fioravanti et al., 2020). Studies have shown that these conserved proteins play an important role as parasites rapidly adapt to different host environments, evade host immune responses, or change their phenotypes during several critical periods (Batugedara et al., 2017).

Recent study has identified a large number of proteins with immunomodulatory activity by comparing the ESP proteomics of three species from *S. mansoni*, *S. japonicum*, and *S. haematobium* and found that the three species have low homology and differences in content (Carson et al., 2020). Further study between these components may identify new proteins including their function or interaction with hosts. In recent studies, a small part of egg-related proteins was found in the ESP of *S. japonicum*, indicating that the ESP can stimulate the innate and adaptive immune system in several different ways. Quantitative SWATH analysis revealed that mature eggs are more likely to stimulate the host immune response than immature eggs (De Marco Verissimo et al., 2019). The bioinformatics analysis of ESPs in schistosomula and adult worms showed that the upregulated differential ESPs in schistosomula were related to stress response, carbohydrate metabolism, and protein degradation, while the upregulated ESPs in adults were mainly related to immune regulation and purine metabolism (Cao et al., 2016b). Actin can form microfilaments with myosin (Reymann et al., 2012) so that the cercariae of *S. japonicum* can better adhere to the muscles during exercise (Mair et al., 2003). Some studies have analyzed the differences between females and males of *Schistosoma* (Cheng et al., 2005; Tallima and El, 2007; Reamtong et al., 2020). It was found that males tend to participate in the actin filamentation process, microtubule process, biosynthesis process, and homeostasis process (Reamtong et al., 2020). Similarly, proteins from males are involved in the proteasome core complex, intracellular signal transduction, and actin polymerization regulation (Tallima and El, 2007). The role of actin in male or various developmental stages is worthy of further study. The binding of praziquantel to *S. japonicum* actin may cause molecular conformational change (Tallima and El, 2007) that severely affects the function of actin-binding proteins and leads to the destruction of worm integrity (Linder and Thors, 1992). Actin may also be a suitable drug target for *S. japonicum*.

Previous studies have focused on *Schistosoma*-derived protein to understand how *Schistosoma* causes damage. *S. japonicum* cercariae invade the host and develop into an immature worm. At this stage, the worm is small and fragile and is an ideal target for host immune attack (Cao et al., 2016a) and is important for early detection. After approximately 3 weeks, the worm grows into an adult, migrates to the portal vein–mesenteric vein system for male and female mating, produces a large number of eggs, and gathers on the liver to form granuloma, causing serious damage to the liver. Eggs can be found in feces at the beginning of 5 weeks. At this time, if treatment is not timely, patients with severe infection begin to develop liver fibrosis and other organ damage that is difficult to alleviate. Due to the phenomenon of immune evasion (McKee and Pearce, 2004) by *S. japonicum*, early detection of *S. japonicum* is particularly difficult, especially in areas with low epidemic levels. *S. japonicum* tegument protein phosphoglycerate mutase (SjPGM) has been identified as a potential disease diagnostic marker (Zhang et al., 2015). Specific IgG antibodies to rSjPGM in the sera of rabbits 2 weeks postinfection increased and then declined gradually from 2 months after praziquantel treatment. The sensitivity and specificity were high, and cross-reactivity of rSjPGM-ELISA in the detection of was low. In *S. haematobium*, Onile et al. (2017) tested and identified potential biomarkers in human urine and identified some potential targets for the diagnosis and treatment of *S. haematobium*. Kardoush et al. (2016) tested and identified potential biomarkers in *S. mansoni* using three mass spectrometry platforms and verified glutathione S-transferase (GST:25KDa) by a Western blot method, which confirmed its potential diagnostic value. Subsequent studies also found that glutathione S-transferase can defend against non-specific oxidation-induced immune attack (Li et al., 2020) of *S. mansoni*. The proteins detected in serum may play an important role in the pathological changes of schistosomiasis. These potential biomarkers may provide a theoretical basis for diagnosis or treatment.

The effect of *S. japonicum* infection and treatment on the host has not previously been studied further. In this study, we selected five time points: non-infection, 1 week and 6 weeks after infection, and 5 weeks and 7 weeks after treatment, and the differential protein began to increase from 1 week after infection, reached the maximum at 6 weeks after infection, and gradually increased with the increase in infection time. After treatment, the differential protein began to decrease with the increase in treatment time, and it was found that the up- and downregulated proteins significantly decreased at 7 weeks after treatment. GO enrichment analyses identified numerous biological processes that are consistent with *Schistosoma* infection and treatment. For example, several biological processes were enriched by proteins that changed during infection or treatment in our study, including receptor-mediated endocytosis, innate immune response, and adaptive immune response. Corresponding to the changes in the protein temporal spectrum in this study, we detected some acute-phase proteins among clusters 5, 6, 7, and 8 that were differentially expressed between the infection and treatment groups, especially the proteins in cluster 7, which can be used as potential biomarkers (**Table 2**). The results showed that compared with the other three groups, the entire protein at 7 weeks after treatment was similar with that of the control group. This is mainly due to the fact that schistosomiasis is dominated by the Th1 immune response at 6–8 weeks and then reaches a peak (Rabello, 1995), and subsequently, a large number of pro-inflammatory cytokines such as tumor necrosis factor, interleukin-1, and interleukin-6 appear (de Jesus et al., 2002). GO analysis also validates this hypothesis because *Schistosoma* infection is immunomodulatory, and therefore, we were not surprised to observe that proteins in the acute inflammatory response were regulated.

**Table 2:** Eighteen host proteins in cluster 7 can be potential biomarkers.

Protein IDs	Protein names	GenBank IDs	Peptide counts	Unique peptides	Sequence coverage [%]	Score
G1TVS4	Hemopexin	AAGW02008019.1;AAGW02008020.1	39	7	69.8	323
G1TFU9	Galectin-3-binding protein		23	23	49.4	323
G1U9R4	Apolipoprotein B	AAGW02055051.1; AAGW02055052.1;AAGW02055053.1;AAGW02055054.1;AAGW02055055.1; AAGW02055056.1;AAGW02055057.1	157	154	45.8	323
A0A5F9D585	Mannan-binding lectin serine peptidase 1	AAGW02014015.1;AAGW02014016.1	23	9	47.7	323
A0A5F9C8F6	Tenascin C		70	70	47.5	323
G1SLB5	Monocyte differentiation antigen CD14	AAGW02027693.1	9	9	36.3	178
A0A5F9CTN2	Transforming growth factor-beta-induced protein ig-h3	AAGW02027826.1;AAGW02027827.1	13	13	30.1	86
P03988	Ig mu chain C region secreted form	K01357.1	19	2	55.9	323
Q865F2	Adhesion molecule VCAM-1	AY212510.1	32	1	59.4	242
Q7M322	Plasmin		1	1	57.6	216
A0A5F9C4W7	SERPIN domain-containing protein	AAGW02039505.1	8	4	29.3	20
G1TV79	Collagen-binding protein	AAGW02008237.1	8	4	50.2	108
G1T0X2	Fibrinogen alpha chain	AAGW02045946.1	24	24	44.9	323
G1T763	Polymeric immunoglobulin receptor	AAGW02025413.1	28	3	50.6	10
G1U7S4	Phosphoglycerate mutase	AAGW02061870.1	5	4	20.6	11
Q6B736	Immunglobulin heavy chain variable region	AY676737.1	3	1	37.1	34
A0A5F9CIB4	Uromodulin	AAGW02057059.1	8	8	14.8	86
A0A5F9C9Q2	Collagen type III alpha 1 chain	AAGW02003526.1	8	8	7.7	92

In the present study, proteins annotated to immune response processes were at the highest levels during infection, with lower levels during treatment. Eight weeks after treatment with praziquantel, the number of eggs greatly decreased, and the disease was in a recovery state (Mnkugwe et al., 2020). The role of these proteins in immune response function requires further investigation but conceivably could be similar to their role in *Schistosoma*-induced infection. The upregulated differential proteins such as immunoglobulin also participate in the immune response in the functional enrichment of GO, and therefore, the increase is more obvious at 6 weeks after infection. Mannan binding lectin serine peptidase 1 (MASP1) in cluster 7 plays a key role in immune function. *Schistosoma* carries glyconjugates at every stage and interacts with mannose-binding lectin (MBL), an innate immune recognition element (Antony et al., 2013). MBL with associated MASP1 and MASP2 interacts with the tegument of *Schistosoma* and activates complement cascades (Klabunde et al., 2000; Antony et al., 2013). The main pathological features of schistosomiasis liver fibrosis are the proliferation of hepatic stellate cells (HSCs) and the deposition of collagen type I (Col I) and collagen type III (Col III) (Chu et al., 2007). The content of collagen increased significantly with the time of infection with *S. japonicum*, and the synthesis of collagen related to liver and granuloma changed to type III collagen (Olds et al., 1985). The dynamic change of collagen type III identified from host proteins was similar to the previous study that up-graduated 1 week after infection and which supposed to be down-graduated after PZQ treatment.

The current study shows that label-free quantitative proteomics is a promising method for identifying antigens involved in schistosomiasis. In our analysis of host differential proteins, we found that the main molecular functions of proteins in the differential analysis groups were protein binding and protease hydrolysis. In the biological process, the main functions were mainly transport, protein hydrolysis, and intracellular immunity, which are related to the acute inflammatory reaction of *S. japonicum* infection. After infection, the high expression of protein binding, hydrolysis, transport, and the metal ion-binding pathway is conducive to protein synthesis to meet the immune needs of the body. We also found that the expression of the many host proteins was decreased after infection, and this may be related to the decrease in the expression of key proteins after severe infection. Our study suggests that additional characteristic molecular changes at protein levels may be used to build a diagnostic model for identification of early cases. Our data elucidate the molecular changes reflected in *Schistosoma* serum, which can potentially yield critical diagnostic markers or therapeutic targets for managing severe *Schistosoma* patients.

Although the genome of *S. japonicum* was sequenced in 2009 and 2019 (*Schistosoma japonicum* Genome and Functional Analysis, 2009; Luo et al., 2019), the function of many proteins is still unknown or unpredicted. The current study is a preliminary examination of the host serum of *Schistosoma japonicum* infection, and the difference analysis and time series analysis of the proteins involved will continue to determine the biomarkers that play a key role in early infection and curative effect assessment. The identified proteins related to *S. japonicum* can be used as markers for the diagnosis of schistosomiasis. In previous reports, these proteins have been used as markers to verify that they can be used as potential biomarkers, and thus, they are reliable for identifying relevant diagnostic or therapeutic markers through a proteome. Serum proteomics is a method used to identify disease biomarkers, and it has been used in many diseases, with many results obtained. As a parasite in blood vessels, *S. japonicum* is still valuable as a satisfactory research subject (Zhong et al., 2017) because of its availability and stability, and the ability to directly observe the changes in its body, although it is difficult to quantify its source protein in the host serum. In this study, the differential proteins produced during the pathological changes of *S. japonicum* were analyzed by comparative proteomics at multiple time points of infection and treatment of *S. japonicum*, which is a feasible method for identifying biomarkers.

There are more than 40 types of natural hosts of *S. japonicum*, among which rabbit is a classical animal model for *S. japonicum* infection with cercariae that is used in a variety of studies (Guo et al., 2012; Huang et al., 2020). Liu et al. (2015) found that the transcriptional profiles of *S. japonicum* in four different hosts were basically the same by comparing the *S. japonicum* gene expression profiles of experimental animal hosts (BALB/c mice, C57BL/6 mice, and rabbits) and natural hosts (water buffalo). In view of the high sensitivity of rabbits to pathogens and the similarity of disease pathology between humans and rabbits (Tran et al., 2020), the New Zealand rabbit was used as the animal model in this experiment, and it was easy to set the time points for infection and treatment and control the intervention conditions. Therefore, it is necessary for a large number of verification tests to be carried out to extend the results to the human body. We focused on host proteins because they play a critical role in repair of infection and cure. However, our study does not capture the complex interactions between host and *Schistosoma*-derived proteins. Proteomics analysis in this study does not serve as an absolute quantification. If the model is to be clinically applied, additional validation of these proteins will be required. The impact of drugs, including praziquantel, on proteomic profiles also requires evaluation. The serum samples were collected at different time points along the disease course, and they can potentially be utilized to explore protein expression changes. Due to the small sample size, future studies on serum from additional time points are required for rigorous temporal analysis.

## Conclusion

Herein, we presented a label-free LC-MS/MS shotgun proteomics approach for biomarkers to distinguish *S. japonicum* infection between the early stage and the course of treatment. This study provides the comprehensive experimental temporal serum proteomics that can be used for early detection of the identified proteins. We identified a number of core factors by GO analysis, and some of them were previously uncharacterized proteins in host proteins. In conclusion, this study presents a systematic proteomic investigation of serum samples from infection groups and treatment groups.

In this work, we found a total of 10 proteins derived from *S. japonicum* and described the expression changes of more than 200 host proteins during five stages of *Schistosoma* infections and treatment. Eighteen host proteins such as mannan-binding lectin serine peptidase 1, immunoglobulin, and collagen can be used as potential biomarkers. These provide a comprehensive landscape of the proteome of *Schistosoma* with direct insight into the protein changes affecting the regulation of the infection process. Our data offer a landscape view of serum protein changes induced by *Schistosoma* infection, which may provide useful diagnostic and therapeutic clues for *S. japonicum*.

## Data Availability Statement

The mass spectrometry proteomics data are available in the ProteomeXchange Consortium *via* the PRIDE partner repository with the dataset identifier PXD029635.

## Ethics Statement

The animal study was reviewed and approved by the Ethical Review Committee of Jiangsu Institute of Parasitic Diseases.

## Author Contributions

KY and G-LY designed the study. N-NB and SZ performed the experiment. SZ and J-FZ conducted the experimental infection of the rabbits. N-NB and YC performed the proteomics assay. N-NB, SZ, and C-YZ conducted the proteomic data analysis. N-NB, KY, and G-LY wrote the article. All authors contributed to the article and approved the submitted version.

## Funding

This research was funded by the grants from the National Natural Science Foundation of China, grant number 82173586, and Jiangsu Provincial Department of Science and Technology, grant number BZ2020003.

## Conflict of Interest

The authors declare that the research was conducted in the absence of any commercial or financial relationships that could be construed as a potential conflict of interest.

## Publisher’s Note

All claims expressed in this article are solely those of the authors and do not necessarily represent those of their affiliated organizations, or those of the publisher, the editors and the reviewers. Any product that may be evaluated in this article, or claim that may be made by its manufacturer, is not guaranteed or endorsed by the publisher.

## Supplementary Material

Supplementary Table 1 GO enrichment of host protein and DEPs.

Supplementary Table 2 Output of proteins identified from *S. japonicum* by MaxQuant.
